# Plasma exchange alone versus combination with intravenous immunoglobulin/methylprednisolone pulse therapy in severe systemic rheumatic diseases: a retrospective study

**DOI:** 10.3389/fimmu.2024.1454691

**Published:** 2024-12-09

**Authors:** Jing Guo, Shuiwen Li, Hao Xu, Xinxin Wang, Weiyuan Luo, Jie Sun, Jianhua Li, Zhu Chen, Guiyang Lu, Xiaolong Huang, Shiju Chen, Yaogui Ning

**Affiliations:** ^1^ Department of Intensive Care Unit, The First Affiliated Hospital of Xiamen University, Xiamen, Fujian, China; ^2^ School of Medicine, Xiamen University, Xiamen, Fujian, China; ^3^ The School of Clinical Medicine, Fujian Medical University, Fuzhou, Fujian, China; ^4^ Department of Rheumatology and Clinical Immunology, The First Affiliated Hospital of Xiamen University, Xiamen, Fujian, China

**Keywords:** systemic rheumatologic diseases, therapeutic plasma exchange, intravenous immunoglobulin, intravenous methylprednisolone pulse, critically ill patients

## Abstract

**Background:**

For severe systemic rheumatic diseases (SRDs), therapeutic plasma exchange (TPE) may be applied as a rescue therapy; it usually combined with intravenous immunoglobulin (IVIG) or intravenous methylprednisolone pulse (IVMP) in severe SRDs. However, the necessity of this combination treatment strategy in SRDs remains uncertain.

**Objective:**

This retrospective study aimed to evaluate the effectiveness of TPE alone versus TPE combined with IVIG/IVMP in treating severe SRDs.

**Methods:**

Patients with severe SRDs treated with TPE who were admitted to the department of intensive care unit (ICU) from January 2011 to December 2019 were included. These patients were divided into two groups: the TPE-alone group (TPE group) and the TPE plus IVIG/IVMP group (TPE + IVIG/IVMP group). The patients’ clinical characteristics, the Sequential Organ Failure Assessment score, the 28-day mortality rate, and the length of ICU stay were evaluated in the two groups.

**Results:**

Ninety-one patients were enrolled in this study: 51 patients in the TPE group and 40 patients in the TPE + IVIG/IVMP group. In the TPE group, the median age was 51.39 ± 15.34 years, and 64.71% were women. In the TPE + IVIG/IVMP group, the median age of the patients was 42.93 ± 16.56 years, and 75% were women. The infection rate in the TPE + IVIG/IVMP group was significantly higher than that in the TPE group (P < 0.05). Both the 28-day mortality and the length of ICU stay did not differ statistically between the two groups (P > 0.05).

**Conclusion:**

This study showed no benefit of combing IVIG/IVMP with TPE in improving the outcome of patients with severe SRDs, suggesting that IVIG/IVMP may not be necessary when used conjunction with TPE for the treatment of severe SRDs.

## Introduction

Systemic rheumatic diseases (SRDs) are characterized by autoimmune mechanisms causing systemic involvement of a tissue or organ; examples of these disorders include scleroderma, polymyositis/dermatomyositis, rheumatoid arthritis (RA), primary Sjögren’s syndrome (pSS), and systemic lupus erythematosus (SLE) (1). The standard therapeutic regime for SRDs includes a variety of immunosuppressive drugs, but not all patients respond well to these immunosuppressive treatments. In some patients, despite immunosuppressive therapy, immune complexes may still form and potentially damage tissues (2). Tissue damage can quickly lead to fatal organ involvement or treatment-related complications, requiring intensive care (2). Therapeutic plasma exchange (TPE) is an adjunctive treatment option for severe SRDs. The mechanism of TPE is based on the removal, for example, of pathogenic antibodies, immune-complexes and cytokines or other macromolecules in the plasma, or, less frequently, albumin-bound small molecules (drugs or toxins) that remain predominantly intravascular (3). This technique can alleviate the pathological process mediated by these pathogenic substances, either by removing pathological factors or by supplementation deficiency ones (4). Some studies are currently exploring the efficacy of steroids combined with TPE for severe SRDs (5, 6), which have shown that lower doses of steroids combined with TPE may reduce the incidence of infections and other complications compared to higher steroid doses alone.

In some cases, severe SRDs often necessitate the combined use of intravenous immunoglobulin (IVIG), typically at 200–400 mg/kg thrice weekly, or intravenous methylprednisolone pulse (IVMP). The main constituent of IVG, immunoglobulin G (IgG), is the major component of IVIG and responsible for the immunomodulatory effects (7). Studies suggested that IVIG’s therapeutic mechanism is marked by peak IgG levels 3 days post-treatment with a half-life lasting up to 30 days (8). The phrase “IVMP” entails swiftly delivering high medication doses via a brief period of time. Methylprednisolone (or dexamethasone in certain regions) is commonly employed as a glucocorticoid.

In cases of severe SRDs, the efficiency of combined therapy of TPE with other drug therapies, specifically IVIG and IVMP, remains ill-defined. These treatments, including IVMP and IVIG as part of the standard of care for SRDs, have unique risk-benefit profiles that necessitate a careful evaluation when paired with TPE. Due to the scarcity of direct comparisons, this retrospective research aimed to assess the efficacy of TPE monotherapy versus its combination with IVIG or IVMP in the management of severe SRDs.

## Methods

### Study population

A retrospective cohort analysis was conducted on patients with severe SRDs admitted to the Department of Intensive Care Unit (ICU) of a large tertiary hospital receiving TPE. Patients who received TPE alone were assigned as the TPE group, whereas those receiving TPE combined with IVIG/IVMP therapy were assigned as the TPE + IVIG/IVMP group. Inclusion criteria: ① Patient diagnosed with SRD. The diagnostic criteria for SLE relied on the latest SLE classification criteria, established by European Alliance of Associations for Rheumatology (EULAR) and American College of Rheumatology (ACR) in 2019, comprising one inclusion criterion, 10 aspects, and 18 criteria. All diagnoses were confirmed through exclusion of infectious, cancerous, medication-induced, and other confounding factors. Each fulfilled historical criteria were scored, with the most severe contributing to the sum scores. A score ≥10 indicated SLE (9). Similarly, EULAR/ACR classification criteria for dermatomyositis (DM), polymyositis (PM), and clinically amyopathic DM (CADM) were applied (10), using 16 variables including clinical manifestations, laboratory measurements, and muscle histology. Antineutrophil cytoplasmic antibody–associated vasculitis (AAV) consists of two main diseases, granulomatosis with polyangiitis and microscopic polyangiitis, ranking among the most severe autoimmune inflammatory disease (11). Signs and symptoms consistent with multiple diseases may arise; however, multisystem involvement is a vital indicator, requiring high suspicion when two or more symptoms are present. Anti-neutrophil cytoplasmic antibody (ANCA) testing is required for those exhibiting potential ANCA vasculitis. This study included patients with both c-ANCA [targeting proteinase 3 (PR3)] and p-ANCA [targeting myeloperoxidase (MPO)] positivity. A clinical diagnosis required both serologic and histologic findings (12). The RA classification requires the presence of at least one swollen joint and 6/10 points from a scoring system (13). Undifferentiated connective tissue disease (UCTD) typically diagnosed by systemic CTD symptoms and laboratory findings but not meeting specific classification criteria (14). Features often include anti-nuclear antibodies positivity, Raynaud’s phenomenon, arthritis/arthralgia, non-specific rash, or sicca symptoms. pSS criteria: A total score of ≥4 calculated by adding up the weight of each positive test/item [focused lymphocytic sialadenitis score of 1, anti-SSA/Ro positive weighted of 3, ocular staining score (OSS) of ≥5 (or van Bijsterveld score (VBS) of ≥4) in at least one eye, Schirmer’s test result ≤ 5 mm/5 min in at least one eye, and an unstimulated whole saliva (UWS) flow rate of ≤0.1 ml/min weighted of 1 each) (15). ② Main indications for TPE included diffuse alveolar hemorrhage, neuropsychiatric involvement (central, peripheral, and psychiatric compromise), respiratory failure, or life−threatening organ dysfunction. In this study, all patients with SRDs were in critical condition, necessitating intensive care. The inclusion criteria focused on patients with severe complications of their SRDs, ensuring that the study group consisted entirely of those requiring aggressive intervention. The patient with RA had undergone TPE due to severe interstitial lung disease, although many treatment strategies (16) including corticosteroids, cyclophosphamide, and mycophenolate had applied but failed to improve the lung involvement. TPE was suggested because of the serious pulmonary involvement, which required a more aggressive therapy beyond standard treatments for active synovitis/arthritis (17). Therefore, TPE have been used in this patient not for the clear indications including rheumatoid vasculitis and hyperviscosity syndrome but as a rescue therapy. ③ Patient admittance to ICU. ④ Each patient received more than three TPE. Exclusion criteria: ① Patient age of <18 years and ② the number of plasmapheresis of <3.

### Data collection and ethics committee approval

For this study, a database was created by reviewing of all available electronic medical records. The following data of the participants were collected: age, sex, Sequential Organ Failure Assessment score (SOFA score) before and after TPE and TPE-related complications. During hospitalization, the following data were recorded: length of ICU stay and 28-day mortality. This study was conducted with the approval of the Institutional Review Board of the Hospital in accordance with the World Medical Association’s Declaration of Helsinki.

### Treatment methods

The TPE procedure was performed daily or on alternate days by continuous flow centrifugation (blood purification devices: Baxter Prismaflex; plasma separator: Prismaflex TPE 1000 set) exchanging at least one calculated volume of plasma per session. Heparin anticoagulation was generally used. Venous access was always a central line, either the right or left femoral vein. Plasma was replaced with the same volume (40–60 mL of plasma/kg) of fresh frozen plasma. Procedures were performed by trained apheresis nurses in the ICU. Treatment was stopped when there was significant improvement and/or death. The TPE group received TPE only.

The IVIG/IVMP group received TPE combined with IVIG, TPE combined with IVIG and IVMP, or TPE combined with IVMP. IVIG was used at a dose of 400 mg/(kg·day) for 3–5 days. IVMP: Patients undertaking IVMP received daily doses ranging from 250 mg to 1,000 mg for 3–5 days, followed by gradual dose reduction. These specific dose and duration were determined by the treating physician considering their condition’s severity and response to therapy. This range covers standard clinical practice and is supported by guidelines and studies (18, 19).

Treatment was stopped when there was significant improvement and/or death.

### Outcomes

The primary endpoint was the 28-day all-cause mortality, and the secondary endpoint was the length of ICU stay.

### Statistical analysis

Descriptive statistics were performed, expressing continuous variables as means ± SD or medians ± interquartile range (IQR). The chi-square or Wilcoxon tests were used to assess the differences, as appropriate. Multiple independent non-normally distributed samples used Kruskal–Wallis test. Fisher’s extract probability test was implemented. Survival curves for patients with and without glucocorticoids were generated via the Kaplan–Meier method and compared using the Gehan–Breslow–Wilcoxon test. Statistical significance was defined as *p* < 0.05. Statistical software SPSS 19 was employed for data analysis.

## Results

### Characteristics of patients with severe SRDs

A total of 91 patients with severe SRDs received TPE were enrolled in this study. Among the 51 patients in the TPE group, the mean age was 51.39 ± 15.34 years, with 64.71% being woman. Their pre-treatment SOFA score averaged 6.08 ± 2.31. Among the 40 patients from the TPE + IVIG/IVMP group, the mean age was 42.93 ± 16.56 years, with 75% being woman. Their pre-treatment SOFA score averaged 6.08 ± 2.31. Significant differences existed between the two groups in terms of age, infection rate, and hematological involvement, with no significant differences in other baseline conditions (**Table 1**).

**Table 1:** Clinical characteristics of the patients before TPE.

Variable	TPE group	TPE+ IVIG/IVMP group	χ^2^/F/Z	*P-value*
N	51	40		
Age, mean ± SD (years)	51.39 ± 15.34	42.93 ± 16.56	2.52	0.013^c^
Female, N (%)	38 (74.51)	30 (75.00)	0.003	0.957^a^
SRD features, N (%)				
SLE	21(41.18)	21 (52.50)	1.157	0.299^a^
DM	9 (17.65)	7 (17.5)	0	1.00^a^
pSS	6 (11.76)	6 (15.00)	0.205	0.759^a^
AAV	7 (13.73)	2 (5.00)	1.95	0.289^b^
CADM	3 (5.88)	3 (7.50)	0.095	1.00^b^
PM	3 (5.88)	0	2.433	0.253^b^
UCTD	1 (1.96)	1 (2.50)	0.03	1.00^b^
RA	1 (1.96)	0	0.793	1.00^b^
Admission to ICU with disease co-morbidities, N (%)				
Infections	7 (13.73)	15 (37.5)	6.91	0.009^a^
Hypertension	11 (21.57)	3 (7.50)	3.41	0.083^b^
Cardiac insufficiency	2 (3.92)	6 (15.00)	3.43	0.132^b^
Diabetes	4 (7.84)	3 (7.50)	0.004	1.00^b^
Cerebrovascular disease	4 (7.84)	3 (7.50)	0.004	1.00^b^
Pregnant	2 (3.92)	3 (7.50)	0.553	0.651^b^
Chronic kidney disease	2 (3.92)	3 (7.50)	0.553	0.651^b^
Viral hepatitis	3 (5.88)	2 (5.00)	0.034	1.000^b^
Tumor	3 (5.88)	0	2.433	0.453^b^
Organ involvement, N(%)				
Renal involvement	7 (13.73)	4 (10.00)	0.293	0.75^b^
Neurologic involvement	6 (11.76)	11 (27.50)	3.654	0.064^a^
Lung involvement	7 (13.73)	3 (7.50)	0.888	0.503^b^
Cardiac involvement	2 (3.92)	2 (5.00)	0.062	1.000^b^
Hepatic involvement	0	1 (2.50)	1.289	0.44^b^
Hematological involvement	2 (3.92)	7 (17.50)	4.638	0.04^b^
Combination treatment N(%)				
None,	51	–	–	–
IVIG	–	25 (62.5)	–	–
IVMP	–	5 (12.5)	–	–
IVIG+IVMP	–	10 (25.00)	–	–
SOFA scores, median (IQR)	6 (4, 7)	7 (5, 10)	1.1	0.271^d^

SLE, systemic lupus erythematosus; DM, dermatomyositis; AAV, antineutrophil cytoplasmic antibody–associated vasculitis; PM, polymyositis; RA, rheumatoid arthritis; CADM, clinically amyopathic dermatomyositis; UCTD, undifferentiated connective tissue disease; pSS, primary Sjögren’s syndrome; IQR, interquartile range; SD, standard deviation. ^a^Pearson chi-square test; ^b^Fisher’s exact test; ^c^Independent sample t-test; ^d^Wilcoxon test; SOFA, Sequential Organ Failure Assessment score; IVIG, intravenous immunoglobulin; IVMP, intravenous methylprednisolone pulse; TPE, therapeutic plasma exchange.

### Risk factors for survival of severe SRDs treated with TPE or TPE + IVIG/IVMP

SOFA scores significantly decreased post-treatment, but no difference exist between the TPE group and the TPE + IVIG/IVMP group (p = 0.08) (**Table 2**). Binary logistic regression analysis models showed that infection and SOFA scores at discharge as critical indicators associated with the risk of patient’s death. To account for the age differences between the groups, a multivariable logistic regression analysis was conducted. To take into consideration any potential confounding effect, age was included to the model as a covariate. Age was eliminated as a predictive factor when utilizing a logistic regression model. The odds ratio (OR) for infection was 9.03, with a 95% confidence interval from 1.32 to 61.58, indicating a notably increased mortality risk among severe SRDs and infection. The findings showed a significant increase in infection risk of poor outcomes (OR = 9.03, P = 0.02) for immunosuppressed patients in critical care settings. Pre- and post-treatment SOFA scores reveal strong corrections with outcomes, and higher post-treatment SOFA scores reveal worse prognosis (OR = 4.78, P < 0.001) (**Table 3**). Logistic regression analysis was used to assess the impact of various factors on infection (**Table 4**). The results of showed that SOFA score at admission was noteworthy predictors of infection, whereas others were not the associated factors.

**Table 2:** Comparison of mean SOFA scores pre-treatment and post-treatment.

	SOFA (pre-treatment), median (IQR)	SOFA (post-treatment), median (IQR)	Z	*P*
TPE group	6 (4,7)	2 (1, 5)	−5.32	0.00
TPE + IVIG/IVMP group	7 (5, 10)	3.5 (2, 7.75)	−3.62	0.00
TPE + IVIG	8 (5, 10.5)	4 (2, 12)	−2.77	0.00
TPE + IVMP	5 (4, 9)	2 (1, 4.5)	−2.03	0.04
TPE + IVIG + IVMP	6 (4, 8)	3.5 (1, 9)	−1.55	0.12
H^a^	0.27	1.41		
*P* ^b^	0.87	0.49		
Z	1.1	1.71		
*P*	0.271	0.08		

TPE, therapeutic plasma exchange; IVIG, intravenous immunoglobulin; IVMP, intravenous methylprednisolone pulse; SOFA, Sequential Organ Failure Assessment score; IQR, interquartile range; ^a^Kruskal–Wallis test was performed for SOFA scores of TPE + IVIG, TPE + IVMP, and TPE + IVIG + IVMP; ^b^The *p*-value represents no difference in SOFA scores between the TPE + IVIG, TPE + IVMP, and TPE + IVIG + IVMP groups before and after treatment.

**Table 3:** Regression models on the effect of treatment on the survival of patients.

Clinical variable	Coefficient	*P*-value	OR (95% CI)
Treatment group	0.23	0.80	1.26 (0.21, 7.44)
Gender	0.69	0.50	1.99 (0.27, 14.51)
Age	0.05	0.18	1.05 (0.98, 1.13)
SRD types	0.90	0.06	2.45 (0.97, 6.23)
Infection	2.20	0.02^*^	9.03 (1.32, 61.58)
SOFA (pre-treatment)	−1.17	0.007^**^	0.31 (0.13, 0.73)
SOFA (post-treatment)	1.56	0.0002^****^	4.78 (2.11, 10.84)

OR, odds ratio; SOFA, Sequential Organ Failure Assessment score; *p < 0.05; **p < 0.01; ****p < 0.0001.

**Table 4:** Regression models of impact on infection.

Variable	Coefficient	P-value	OR (95% CI)
Treatment group	1.2867	0.018*	3.62 (0.22, 2.35)
Gender	−0.0607	0.918	0.94 (−1.21, 1.09)
Age	0.0273	0.104	1.03 (−0.01, 0.06)
SRD types	0.1452	0.611	0.86 (−0.70, 0.41)
SOFA (pre-treatment)	0.2614	0.021*	1.30 (0.04, 0.48)
SOFA (post-treatment)	−0.0093	0.908	0.99 (−0.17, 0.15)

OR, odds ratio; SOFA, Sequential Organ Failure Assessment score; *p < 0.05.

### Outcomes of the patient

**Figure 1:**
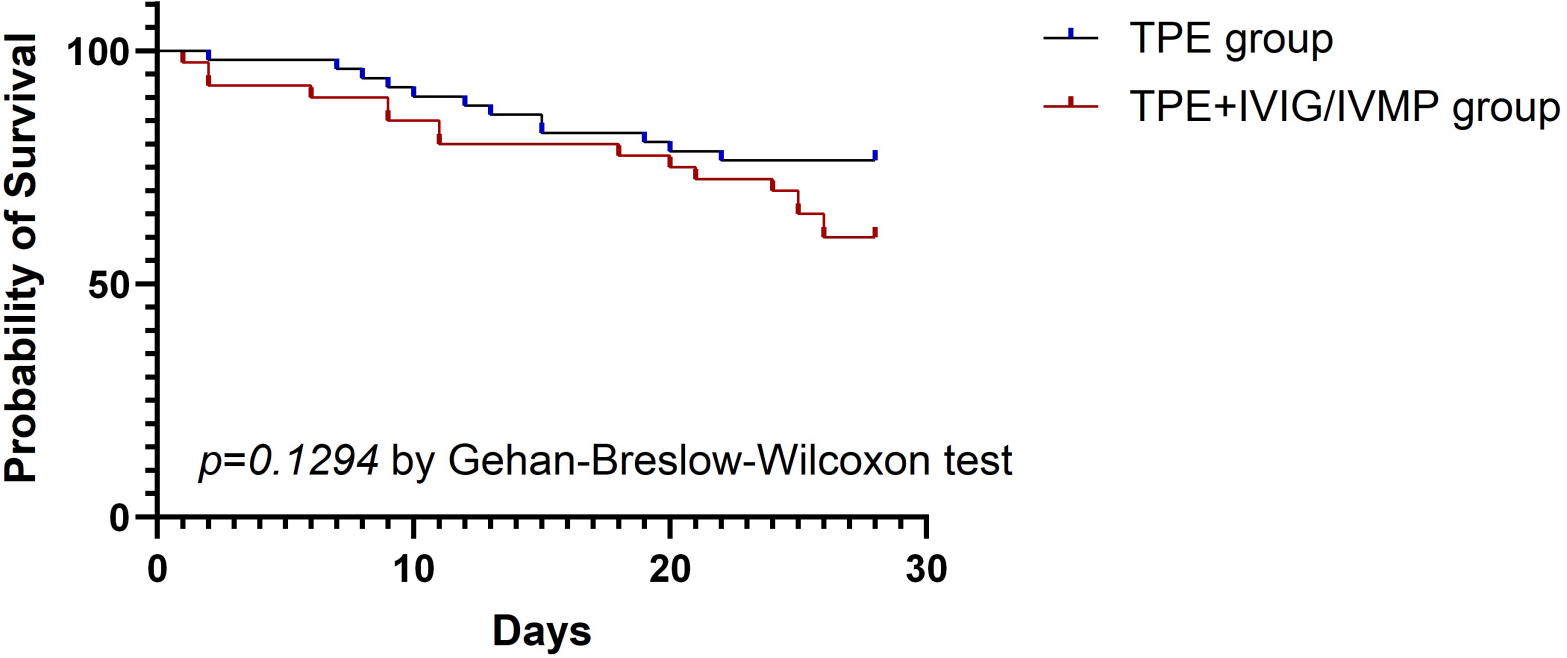
Kaplan–Meier survival analysis.

The 28-day all-cause mortality rate was 40% in the TPE + IVIG/IVMP group and 23.53% in the TPE-alone group. The length of ICU stay was 15.38 ± 12.33 in the TPE + IVIG/IVMP group and 13.25 ± 12.11 in the TPE-alone group. No significant differences were found between the two groups (*P* > 0.05) (**Table 5**). The 28-day all-cause mortality rate and the length of ICU stay were analyzed independently for the TPE and TPE + IVIG groups in **Table 6**. P-values suggest that there are no statistically significant differences. Likewise, patient survival analysis via Kaplan–Meier curves revealed no notable survival differences between the TPE and TPE + IVIG/IVMP cohorts (P = 0.1294) (**Figure 1**).

**Table 5:** Outcomes of the patients with different treatments.

	TPE group	TPE + IVIG/IVMP group	*P-*value
28-day all-cause mortality, n (%)	12 (23.53)	16 (40.00)	0.13
Length of ICU stay, mean ± SD (days)	13.25 ± 12.11	15.38 ± 12.33	0.41

TPE, therapeutic plasma exchange; IVIG, intravenous immunoglobulin; IVMP, intravenous methylprednisolone pulse; ICU, intensive care unit.

**Table 6:** Subgroup analysis: TPE group vs. TPE + IVIG group.

	TPE	TPE + IVIG	*P-*value
28-day all-cause mortality, n (%)	12 (23.53)	11 (44.00)	0.068
Length of ICU stay, mean ± SD (days)	13.25 ± 12.11	16.20 ± 12.16	0.323

TPE, therapeutic plasma exchange; IVIG, intravenous immunoglobulin; ICU, intensive care unit.

## Discussion

This 8-year retrospective analysis included 91 patients with severe SRDs admitted to the ICU treated with TPE. The results demonstrated no significant advantage of combining IVIG/IVMP with TPE over TPE alone for patients with severe SRDs. We observed a significant improvement in SOFA scores post-treatment, yet no group-wise difference, indicating equivalent treatment efficacy in both groups. Both groups exhibited no significant differences in 28-day mortality and length of ICU stay. However, the addition of IVIG/IVMP failed to improve patient outcomes. Despite no significant difference in mortality, the TPE-alone group’s mortality rate (23.53%) was lower than that in non-TPE studies (25%–55%) (20). The IVIG/IVMP + TPE group’s mortality rate (40.00%) was higher than that in the TPE-alone group (23.53%), although statistically insignificant. Larger studies are needed in the future. Autoimmune diseases encompass a broad spectrum of clinical conditions, primarily involving multiple organ systems. Autoantibodies are the primary pathogenic factors. Hence, the rationale for TPE in autoimmune diseases, especially during acute, life-threatening phases and when immunosuppressive therapy is ineffective, is very strong. The mechanism of TPE involves the removal of classical or canonical antibodies, which is the basis for its use in vasculitis. Additional benefits come from the removal of lesser known antibodies or substances such as anti–lysosome-associated membrane protein-2 (LAMP-2) antibodies, coagulation factors, complement derivatives, and adhesion molecules (21). This process may also mitigate the inflammatory cascade and the subsequent sequelae. Our data indicate that the adjunctive use of IVIG/IVMP in the acute phase does not provide overall benefits. While many studies advocate TPE in conjunction with IVMP/IVIG for severe SRD cases (22, 23), our study suggests that the complementary use of IVIG/IVMP to TPE in the acute phase offers no additional benefits. All included cases suffered from severe, life-threatening symptoms of SRDs, necessitating aggressive treatment. This study aimed to evaluate the efficacy of TPE and IVIG across a range of severe autoimmune conditions, requiring intensive immunosuppressive therapy. The analysis indicated that the distribution of various immune diseases was comparable between the TPE and TPE + IVIG/IVMP groups, minimizing potential bias from grouping diverse SRDs together (**Table 1**). This consistency validates our findings. Future research with more precise disease stratification may provide additional insights into the varying impacts of TPE and IVIG across various autoimmune disorders. By focusing on patients with severe SRDs, we aim to provide a clearer understanding of the efficacy of these treatments in managing life-threatening complications.

Notably, despite the widespread use of high-dose glucocorticoids in SRD treatment, their definitive therapeutic impact remains elusive. IVMP is the primary treatment method for severe and (or) life-threatening SLE. Methylprednisolone binds to glucocorticoid receptor (GR) to exert anti-inflammatory effect via regulating leukocyte migration, immune cell activation, and proinflammatory cytokine production (24). High-dose methylprednisolone triggers lymphocyte apoptosis, contributing to the immunosuppressive mechanism of IVMP (25). IVMP promoted CD4+ T-cell apoptosis, leading to macrophage production of transforming growth factor-β (TGF-β). Elevated TGF-β promotes Treg differentiation, suppressing CD4+ T-cell activation and proliferation, and ultimately fostering an immunoregulatory milieu induced by IVMP (26). Dexamethasone, due to its enhanced GR affinity and lower protein binding, exhibits greater anti-inflammatory activity compared to methylprednisolone (27). Methylprednisolone may offer quicker cellular penetration advantages over dexamethasone (28). Following liver conversion into pharmacologically active prednisolone, the drug provides immediate and profound anti-inflammatory effects with reduced toxicity compared to a higher dose oral regimen. This approach results in faster symptom resolution than oral therapy, thus reducing inflammation damage. Clinical improvement persist for approximately 3 weeks post-pulse, lacking long-term hypothalamic–pituitary axis suppression (29). Some studies suggest that plasma exchange increase the rate of renal recovery in ANCA-associated systemic vasculitis presenting with renal failure when compared to intravenous methylprednisolone; however, intravenous methylprednisolone was associated with a greater risk of infection and diabetes (6, 30). Plasma exchange and glucocorticoid dosing in the treatment of anti–neutrophil cytoplasm antibody–associated vasculitis indicated that routine use of plasma exchange alongside with high-dose glucocorticoid infusion did not enhance long-term kidney recovery and patient survival (5). Although intravenous pulse glucocorticoid administration can lead to potentially severe complications, such as tachycardia (13.3%), hypertension (8.3%), headache (1.7%), and flushing (1.7%) (31). Hyperglycemia, hypokalemia and infections are also common adverse effects. Higher cumulative methylprednisolone doses (>5 g) increase infection risk (32). Our study’s combination of IVIG/IVMP with TPE did not show additional clinical benefits and increased the risk of infection. The infection rate in the IVIG/IVMP with TPE group was higher than that in the TPE-alone group, indicating a significant difference (*P* < 0.05). Our logistic regression analysis confirmed a statistically significant infection association, meaning its relevance to patient mortality risk. The IVMP dose range (250–1,000 mg/day) was personized to each patient’s condition and response. The different doses may impact side effects and mortality; however, the variability in doses enhances the generalizability of our findings in real clinical settings. **Table 1** shows a higher proportion of IVMP use in patients with neurologic involvement than those with other organ involvements, which could potentially affect study outcomes. Future studies with larger cohorts and balanced representation of different organ involvements would help confirm this.

IVIG, a polyclonal antibody biomolecule, composed mainly of IgG and minor quantities of immunoglobulin M (IgM) and immunoglobulin A (IgA), is derived from plasma banks (33). Its mechanism involves modulating the expression and function of Fc receptors, interfering with complement activation and the cytokine networks, offering of anti-idiotypic antibodies and modulating T- and B-cell response (34). The immune globulin’s therapeutic efficacy is probably due to the natural antibodies’ roles in preserving immunological homeostasis in healthy individuals. So far, IVIG treatment remains off-label for patients with autoimmune disease by the FDA, yet it has proven beneficial and safe in numerous conditions such as SLE, AAV, catastrophic antiphospholipid syndrome, and pSS (35).

IVIG has been reported to be beneficial in various SLE presentations or resistant cases. Occasionally, IVIG is used as a primary treatments for patients who refuse immunosuppressants or those with concurrent systemic infections or neurological impairments (36, 37). IVIG regulates immunity, but plasmapheresis reduces immunoglobulins—IgE, IgG, IgM, and IgA—by more than 40% (38), suggesting that therapeutic plasma exchange could be less expensive than polyvalent immunoglobulin from a healthcare perspective (39). Although immunoglobulin is well tolerated, adverse effects do occur, mostly mild and resolved post-infusion cessation, but severe side effects like aseptic meningitis, renal impairment, thrombosis, and hemolytic anemia can also occur (40). The elevated infection rates observed in the TPE + IVIG/IVMP group might be resulted from several reasons: Firstly, some patients also engaged in other immunosuppressive therapies like IVMP concurrent with IVIG. The combined immunosuppressive effect of these therapies can further increase the susceptibility to infections. Secondly, the elevated infection rate in the IVIG group could potentially stem from these patients’ preexisting infection at admission. The prescription of IVIG combined with TPE the was based on physicians’ clinical judgment for individual patient’s conditions including disease severity, complications, and initial treatment responses. However, the analysis failed to show the advantages of adding IVIG in improving the outcomes in most cases, which suggested that IVIG might not be essential for many patients receiving TPE. The present study just provided some valuable real-world experience into the necessity of combining IVIG with TPE. Future more well-designed prospective studies are needed to compare the TPE plus IVIG with solely TPE. During TPE, IVIG/IVMP may be unnecessary, potentially increasing infection risk and filtering the propyl sphere during plasma exchange, thereby increasing medical cost. According to our research, these patients may be treated with fewer immunosuppressive medications, which could help reduce the risk of infection. Optimizing immunosuppressive therapy, possibly reducing corticosteroid use during TPE, may decrease infection risk for critically ill immunosuppressed patients. Together with comprehensive infection prevention, this method could improve patients’ outcomes.

Different SRD subtypes exhibit significant pathophysiological heterogeneity, which may lead to varied patient responses to combined IVIG/IVMP and TPE therapy. During acute episodes, TPE can directly remove pathogenic antibodies, immune complexes, and cytokines from circulation, making it effective for rapid inflammation control. In contrast, the mechanisms of IVIG/IVMP are relatively slower, involving Fc receptor modulation, complement inhibition, and the regulation of T-cell and B-cell immune responses, which may not have immediate effects during acute phases. This difference in mechanisms could contribute to the lack of observed benefit from adjunctive therapy in our study. Additionally, the pathophysiological differences between SRD subtypes may influence responses to combination therapy; thus, future research could benefit from analyzing these therapies effects in more specific subpopulations to further elucidate the potential role of IVIG/IVMP in SRD treatment.

Despite our findings providing important insights in the use of TPE and IVIG/IVMP for treating SRDs, certain limitations persist. Firstly, as the nature of retrospective study design, selection bias could not be avoided as difference like age existed between the two groups. Secondly, this study was conducted in a tertiary hospital, which might lack generalizability due to SRD-specific centers. Lastly, single center and small sample size of this study limit the generalizability and statistical power of our findings, and results should be interpreted with caution. Hence, multi-center, prospective studies are needed to validate these findings and produce more universally applicable evidence for a larger patient demographic. Moreover, the broad spectrum and heterogeneity of autoimmune diseases pose a challenge in tailoring treatment strategies, and our findings underscore the necessity for a nuanced understanding of how different SRD subtypes respond to TPE, with or without the adjunctive use of IVIG/IVMP. Future research should aim to delineate therapeutic effects across various SRD subtypes, considering factors such as disease severity, activity levels, and organ involvement. These limitations compromised the generalizability and statistical power of our findings, and the results should be interpreted with caution. In future, large-scale, prospective, multicenter studies with rigorous control for confounders and standardized treatment protocols are needed to further validate our findings.

## Conclusion

TPE + IVIG/IVMP did not improve the prognosis of patients with severe SRDs; therefore, there was no need for IVIG/IVMP during TPE when treating this subgroup. This study provides preliminary clinical insights into managing critically ill patients with SRD in ICU settings. However, due to its single-center, small-sample, and retrospective nature, these findings should be interpreted with caution. Future large-scale, prospective, multicenter studies with rigorous control for confounders and standardized treatment protocols are needed to further confirm our findings. Without more evidence, we suggest cautious use of adjunctive IVIG/IVMP with TPE for patients with SRD in ICU settings.

## Data Availability

The original contributions presented in the study are included in the article/supplementary material. Further inquiries can be directed to the corresponding authors.

## References

[1] Pepmueller PH. Undifferentiated connective tissue disease, mixed connective tissue disease, and overlap syndromes in rheumatology. Mo Med. (2016) 113:136–40. PMC6139943 27311225

[2] Arjmand M Shahriarirad R Shenavandeh S Fallahi MA-O. Determination of the main causes, outcome, and prognostic factors of patients with rheumatologic diseases admitted to the medical intensive care unit in Southern Iran. Clin Rheumatol. (2022) 41:3859–68. doi: 10.1007/s10067-022-06334-5 PMC9376566 35969279

[3] Winters JL. Plasma exchange: concepts, mechanisms, and an overview of the American Society for Apheresis guidelines. Hematol Am Soc Hematol Educ Progr. (2012) 2012:7–12. doi: 10.1182/asheducation-2012.1.7 23233554

[4] Clark WF Rock GA Buskard N Shumak KH LeBlond P Anderson D. Therapeutic plasma exchange: an update from the Canadian Apheresis Group. Ann Intern Med. (1999) 131:453–62. doi: 10.7326/0003-4819-131-6-199909210-00011 10498563

[5] Walsh M Merkel PA Peh CA Szpirt WM Puéchal X Fujimoto S. Plasma exchange and glucocorticoids in severe ANCA-associated vasculitis. N Engl J Med. (2020) 382:622–31. doi: 10.1056/NEJMoa1803537 PMC7325726 32053298

[6] Cheng L Gou SJ. Whether the addition of high-dosage methylprednisolone to plasma exchange was more effective than plasma exchange in the treatment for severe antineutrophil cytoplasmic antibody-associated vasculitis? Kidney Int. (2022) 101:647–8. doi: 10.1016/j.kint.2021.11.025 35190039

[7] Dalakas MC. Intravenous immune globulin therapy for neurologic diseases. Ann Intern Med. (1997) 126:721–30. doi: 10.7326/0003-4819-126-9-199705010-00008 9139559

[8] Dalakas MC. Mechanisms of action of IVIg and therapeutic considerations in the treatment of acute and chronic demyelinating neuropathies. Neurology. (2002) 59:S13–21. doi: 10.1212/wnl.59.12_suppl_6.s13 12499466

[9] Aringer M CK Daikh D Brinks R Mosca M Ramsey-Goldman R Smolen JS. 2019 European league against rheumatism/American college of rheumatology classifcation criteria for systemic lupus erythematosus. Ann Rheumatic Dis. (2019) 78:1151–9. doi: 10.1136/annrheumdis-2018-214819 31383717

[10] Lundberg IE Tjärnlund A Bottai M Werth VP Pilkington C Visser M. 2017 European League Against Rheumatism/American College of Rheumatology classification criteria for adult and juvenile idiopathic inflammatory myopathies and their major subgroups. Ann Rheumatic Dis. (2017) 76:1955–64. doi: 10.1136/annrheumdis-2017-211468 PMC5736307 29079590

[11] Kronbichler A Bajema IM Bruchfeld A Mastroianni Kirsztajn G Stone JH. Diagnosis and management of ANCA-associated vasculitis. Lancet. (2024) 403:683–98. doi: 10.1016/S0140-6736(23)01736-1 38368016

[12] Hunter RW Welsh N Farrah TE Gallacher PJ Dhaun N. ANCA associated vasculitis. Bmj. (2020) 369:m1070. doi: 10.1136/bmj.m1070 32291255 PMC7179255

[13] Aletaha D Neogi T Silman AJ Funovits J Felson DT Bingham CO 3rd. 2010 Rheumatoid arthritis classification criteria: an American College of Rheumatology/European League Against Rheumatism collaborative initiative. Ann Rheumatic Dis. (2010) 69:1580–8. doi: 10.1136/ard.2010.138461 20699241

[14] Rubio J Kyttaris VA-O. Undifferentiated connective tissue disease: comprehensive review. Curr Rheumatol Rep. (2023) 25:98–106. doi: 10.1007/s11926-023-01099-5 36884206

[15] Shiboski CH Shiboski SC Seror R Criswell LA Labetoulle M Lietman TM. 2016 American college of rheumatology/European league against rheumatism classification criteria for primary sjögren’s syndrome: A consensus and data-Driven methodology involving three international patient cohorts. Arthritis Rheumatol. (2016) 69:35–45. doi: 10.1002/art.39859 27785888 PMC5650478

[16] Hallowell RW Horton MR. Interstitial lung disease in patients with rheumatoid arthritis: spontaneous and drug induced. Drugs. (2014) 74:443–50. doi: 10.1007/s40265-014-0190-z 24570384

[17] Akiyama M Kaneko Y. Pathogenesis, clinical features, and treatment strategy for rheumatoid arthritis-associated interstitial lung disease. Autoimmun Rev. (2022) 21:103056. doi: 10.1016/j.autrev.2022.103056 35121155

[18] Smolen JS Landewé R Bijlsma J Burmester GR Dougados M Kerschbaumer A. EULAR recommendations for the management of rheumatoid arthritis with synthetic and biological disease-modifying antirheumatic drugs: 2016 update. Ann Rheum Dis. (2017) 76:960–77. doi: 10.1136/annrheumdis-2016-210715 28264816

[19] Hahn BH McMahon MA Wilkinson A Wallace WD Daikh DI Fitzgerald JD. American College of Rheumatology guidelines for screening, treatment, and management of lupus nephritis. Arthritis Care Res (Hoboken). (2012) 64:797–808. doi: 10.1002/acr.21664 22556106 PMC3437757

[20] Quintero OL Rojas-Villarraga A Mantilla RD Anaya JM. Autoimmune diseases in the intensive care unit. An update. Autoimmun Rev. (2013) 12:380–95. doi: 10.1016/j.autrev.2012.06.002 22743032

[21] Casian A Jayne D. Plasma exchange in the treatment of Wegener’s granulomatosis, microscopic polyangiitis, Churg-Strauss syndrome and renal limited vasculitis. Curr Opin Rheumatol. (2011) 23:12–7. doi: 10.1097/BOR.0b013e32834120c1 21124082

[22] Ruffatti A Favaro M Brucato A Ramoni V Facchinetti M Tonello M. Apheresis in high risk antiphospholipid syndrome pregnancy and autoimmune congenital heart block. Transfus Apher Sci. (2015) 53:269–78. doi: 10.1016/j.transci.2015.11.006 26626966

[23] Matsui M Okuma Y Yamanaka J Uryu H Sato N Shichino H. Kawasaki disease refractory to standard treatments that responds to a combination of pulsed methylprednisolone and plasma exchange: Cytokine profiling and literature review. Cytokine. (2015) 74:339–42. doi: 10.1016/j.cyto.2015.02.014 25801094

[24] Stahn C Buttgereit F. Genomic and nongenomic effects of glucocorticoids. Nat Clin Pract Rheumatol. (2008) 4:525–33. doi: 10.1038/ncprheum0898 18762788

[25] Migita K Eguchi K Kawabe Y Nakamura T Shirabe S Tsukada T. Apoptosis induction in human peripheral blood T lymphocytes by high-dose steroid therapy. Transplantation. (1997) 63:583–7. doi: 10.1097/00007890-199702270-00017 9047155

[26] Sun JL Lyu TB Chen ZL Lian CF Liu SY Shao TH. Methylprednisolone pulse therapy promotes the differentiation of regulatory T cells by inducing the apoptosis of CD4(+) T cells in patients with systemic lupus erythematosus. Clin Immunol. (2022) 241:109079. doi: 10.1016/j.clim.2022.109079 35842211

[27] Schimmer B. Adrenocorticotropic hormones, adrenocortical steroids and their synthetic analogues; inhibitors of the synthesis and actions of adrenocortical hormones. In: Goodman & Gilman’s The Pharmacological Basis of Therapeutics, 9th ed. New York, NY: McGraw-Hill (1996).

[28] Wilson J. Cellular localization of 3H-labelled corticosteroids by electron microscopic autoradiography after hemorrhagic shock. In: Steroids and Shock. Baltimore: University Park (1974), 275–99.

[29] Novak E Stubbs SS Seckman CE Hearron MS. Effects of a single large intravenous dose of methylprednisolone sodium succinate. Clin Pharmacol Ther. (1970) 11:711–7. doi: 10.1002/cpt1970115711 4917091

[30] Jayne DR Gaskin G Rasmussen N Abramowicz D Ferrario F Guillevin L. Randomized trial of plasma exchange or high-dosage methylprednisolone as adjunctive therapy for severe renal vasculitis. J Am Soc Nephrol. (2007) 18:2180–8. doi: 10.1681/ASN.2007010090 17582159

[31] Aghighi Y Attarod L Javanmard M. Efficacy of methylprednisolone pulse therapy in children with rheumatoid arthritis. Clin Rheumatol. (2008) 27:1371–5. doi: 10.1007/s10067-008-0919-8 18604580

[32] Kang I Park SH. Infectious complications in SLE after immunosuppressive therapies. Curr Opin Rheumatol. (2003) 15:528–34. doi: 10.1097/00002281-200309000-00002 12960476

[33] Katz U Achiron A Sherer Y Shoenfeld Y. Safety of intravenous immunoglobulin (IVIG) therapy. Autoimmun Rev. (2007) 6:257–9. doi: 10.1016/j.autrev.2006.08.011 17317619

[34] Bayry J Misra N Latry V Prost F Delignat S Lacroix-Desmazes S. Mechanisms of action of intravenous immunoglobulin in autoimmune and inflammatory diseases. Transfusion Clinique Biologique. (2003) 10:165–9. doi: 10.1016/s1246-7820(03)00035-1 12798851

[35] Katz U Shoenfeld Y Zandman-Goddard G. Update on intravenous immunoglobulins (IVIg) mechanisms of action and off- label use in autoimmune diseases. Curr Pharm Des. (2011) 17:3166–75. doi: 10.2174/138161211798157540 21864262

[36] Wang J McQuilten ZK Wood EM Aubron C. Intravenous immunoglobulin in critically ill adults: When and what is the evidence? J Crit Care. (2015) 30:652.e9–.e6.52E16. doi: 10.1016/j.jcrc.2015.01.022 25702845

[37] Harvey RD 3rd. The patient: Emerging clinical applications of intravenous immunoglobulin. Pharmacotherapy. (2005) 25:85S–93S. doi: 10.1592/phco.2005.25.11part2.85S 16229679

[38] Lambrecht L Vanholder R Ringoir S. Single-needle membrane plasmapheresis. *In vivo* comparison of plasma separator performances. Blood Purif. (1988) 6:77–84. doi: 10.1159/000169487 3395473

[39] Moranne O Roux C Ion IM Chkair S. Therapeutic plasmapheresis procedures: An alternative to the disruption of the supply of polyvalent immunoglobulin in autoimmune pathologies. Medico-economic study. Nephrol Ther. (2022) 18:172–9. doi: 10.1016/j.nephro.2022.02.004 35644771

[40] Guo Y Tian X Wang X Xiao Z. Adverse effects of immunoglobulin therapy. Front Immunol. (2018) 9:1299. doi: 10.3389/fimmu.2018.01299 29951056 PMC6008653

